# Overcoming financial limitations in global volcano monitoring

**DOI:** 10.1038/s41467-021-22247-4

**Published:** 2021-04-07

## Abstract

Equitable partnerships among the international volcano science community are important now more than ever, to cope with financial disparities and ultimately allow for worldwide volcano monitoring oriented to hazard mitigation.

Volcanoes pose a broad range of threats, spanning from lava flows to ash fall, lahars (mudflows), and the release of toxic gases. Despite the potential hazards of volcanoes, their immediate environments are often heavily populated and tourist magnets. Sadly, the high unpredictability of volcanic activity and the immense difficulty of forecasting eruptions can lead to loss of life. In December 2019 the unexpected eruption of Whakaari/White Island in New Zealand resulted in 21 fatalities^1^. Only months before that, in July 2019, Stromboli Volcano in Italy caused one fatality, again as a result of an unexpected violent eruption^2^. The densely populated and immediate environments of active volcanoes call for caution and accurate monitoring of volcanic activity in order to evaluate risk.

More networks like these are needed to allow for further, active exchange of knowledge and experience within the global volcanology community

Effective volcano monitoring pursues two main goals: the acquisition of data to better understand how volcanoes work and the use of such data to forecast imminent eruptions with an adequate warning time to protect nearby populations. Some of the most useful monitoring information are seismic, degassing, and ground deformation data. Monitoring in the past decades has been largely facilitated by the continuous advance of ground-, air-, and space-based monitoring technologies, and has allowed the scientific community to make a big leap forward towards more accurate predictions of volcanic eruptions. However, access to some of this technology can often be limited by the high costs (e.g. up to several tens of thousands of USD for single hardware pieces such as seismometers, and up to several hundreds of thousands of USD for entire networks), and therefore is not financially feasible for some governments, in particular of low-income countries. This in turn leaves a large portion of the estimated 1500 active volcanoes worldwide monitored insufficiently and the population in their vicinity at increased risk (https://volcano.si.edu/).

Seeing this as a key motivation to study pathways of low-cost volcano monitoring, a number of technical developments, such as unmanned aerial vehicles (UAVs or drones), have been increasingly touted as a viable solution in recent years. Costing only a few hundred USD and equipped with a range of sensors and cameras, they can monitor topographical changes, analyze or collect in situ gas samples from the plume or investigate the thermal structure of a volcano—all data that gives critical feedback on its activity status^3,4^. Another advantage is the mobility of drones, as they allow for remote access of potentially highly dangerous areas such as the inside of craters or the close-up investigation of volcanic conduits. Low-cost monitoring of active volcanoes, e.g. via drones, may not achieve the same data quantity output as top-tier monitoring instrumentation networks installed at volcanoes such as, e.g., Etna (Italy), Kīlauea (USA), or Soufrière Hills (Montserrat). However, it can do an excellent job in monitoring the activity status of volcanoes and must not be considered low-quality data. Each piece of volcanic unrest information may contribute to a more adequate warning time prior to an imminent eruption, which serves the ultimate goal—risk mitigation for the population.

Evaluation of risk from volcanic activity does not only rely on the acquisition of activity data but also on its interpretation. In 2015, Latin American Volcanologists published a summary of their most pressing challenges in volcanic risk reduction, and highlighted not only a lack of financial and technical resources but also human resources^5^. With human expertise critical for data analysis and interpretation there have been many successful examples of partnerships and knowledge exchange between international universities, agencies, and observatories over the years. Parachute science must be avoided at all costs, however. Local scientists can take advantage of such partnerships, but must have complete agency in how they monitor their own volcanoes independently from nations in Europe and North America.

Along these lines, the 2011–2015 EU-funded VUELCO project (https://vuelco.net) is an example of how such equitable partnerships can work. Volcanic unrest simulation exercises were conducted at various locations, such as Cotopaxi, Colima and Dominica (South and Central America), and Campi Flegrei in Italy (Europe), bringing together local and international science communities, decision-makers, and disaster-response agencies. Regional emergency plans were tested allowing disaster response teams to evaluate their emergency protocols and prepare local volcanologists and decision-makers for the case of a future event. Most importantly, it allowed for an active debate between global volcano communities, exchange of experience, and the possibility to learn from one another. Another example is the STREVA project (https://streva.ac.uk/) which is a partnership between local agencies and international universities that aims to analyse volcanic risk and hazard, and to find ways to effectively communicate this risk to the local population to strengthen resilience.

More networks like these are needed to allow for further, active exchange of knowledge and experience within the global volcanology community. This could be one major step towards making more countries fully self-sustained in monitoring their own volcanoes. It should be noted that even the best monitored volcanoes can behave unpredictably leading to loss of life. But equitable and inclusive partnerships combined with low-cost monitoring techniques will produce better data with stronger predictive power, bringing us closer to the foremost goal of risk reduction at populated and active volcanoes.
